# Replication-competent adenovirus reporters utilizing endogenous viral expression architecture

**DOI:** 10.1128/jvi.01146-25

**Published:** 2025-09-10

**Authors:** Claire M. O’Brien, Lorenzo Serra, Molly R. Patterson, Reyes W. Acosta, Alison Yu, Daniel T. Claiborne, Alexander M. Price

**Affiliations:** 1 Genome Regulation and Cell Signaling, Ellen and Ronald Caplan Cancer Center, The Wistar Institute 351209 , Philadelphia, Pennsylvania, USA; 2 Department of Pharmacy and Biotechnology, Cellular and Molecular Biology Program, University of Bologna 9296, Bologna, Italy; 3 Cell and Molecular Biology Graduate Group, Perelman School of Medicine, University of Pennsylvania 6572, Philadelphia, Pennsylvania, USA; Tufts University School of Medicine, Boston, Massachusetts, USA

**Keywords:** infectious unit quantification, live-cell imaging, viral replication kinetics, bioluminescent virus, fluorescent reporter virus, reporter virus, adenovirus

## Abstract

Adenoviruses are double-stranded DNA viruses widely used as platforms for vaccines, oncolytics, and gene delivery. However, tools for studying adenoviral gene expression in real time during infection remain limited. Here, we describe a set of fluorescent and bioluminescent reporter viruses built using the modular AdenoBuilder reverse genetics system and informed by high-resolution maps of Ad5 transcription. These reporters utilize endogenous early and late transcriptional units, enabling visualization of viral gene expression without exogenous promoters or splicing elements. These model viruses replicate with kinetics nearly indistinguishable from wild-type virus and have enabled real-time fluorescent imaging as well as longitudinal bioluminescent sampling from the same infected samples. Together, this next generation of adenovirus reporters provides a modular and tractable platform for studying viral gene regulation and replication dynamics in real time, with broad applications for basic research and high-throughput screening.

**IMPORTANCE:**

This research provides powerful new tools to rapidly study adenovirus gene expression and replication. By integrating fluorescent and secreted luciferase reporters into native viral regulatory elements, we enable real-time, non-destructive tracking of early and late stage infection in living cells. These modular reporters are compatible with a wide range of genetic and chemical perturbations, allowing researchers to investigate the function of specific viral genes, host interactions, and the impact of host genes and antiviral compounds. Importantly, the high-throughput nature of these systems overcomes limitations of traditional plaque assays to quantify viral replication dynamics. Our work will allow for the rapid creation of both novel infectious and replication-incompetent viral vectors.

## INTRODUCTION

Adenoviruses (AdV) are prevalent pathogens of both humans and non-human animals (1). Human AdV belongs to over 100 different serotypes, spread over 7 major clades (A–G). AdV infection can cause a wide range of diseases and clinical manifestations, typically clustering with viral clade, that include acute respiratory infection, conjunctivitis outbreaks, and gastroenteritis (2). While often self-limiting, infections in immunocompromised individuals can be devastating, with systemic infection being associated with high mortality (3). Furthermore, even in otherwise healthy individuals, AdV can have rare but life-threatening consequences (4). These include viral hepatitis or severe diarrhea in pediatric populations (5–7), as well as epidemic outbreaks of severe acute respiratory distress in young adults (8). Despite the obvious impact on human health, there are no approved or effective antivirals specifically targeting AdV infection, nor are there any vaccines for civilian use.

For many years, replication-incompetent AdV vectors have been widely used for vaccines, oncolytic therapies, gene delivery, and basic research. Notably, several licensed vaccines employing these vectors were rapidly deployed during the SARS-CoV-2 pandemic (9, 10). AdV vectors offer advantages over other viral platforms, including a relatively large packaging capacity, high transduction efficiency, and the ability to infect both dividing and non-dividing cells (11). However, deletions made to increase cargo capacity can impair viral replication and must be complemented in producer cell lines to enable efficient packaging. In addition, genomes that are too small or too large are poorly packaged into the ordered icosahedral capsid, complicating the design of both replication-competent and -incompetent vectors (12). Challenges such as re-targeting vectors to novel cell types and overcoming widespread pre-existing immunity to common AdV serotypes remain major hurdles in adenoviral vector engineering.

AdV has served as a powerful and tractable model system, leading to numerous seminal discoveries in both virology and cell biology. It was among the first viruses shown to have oncogenic potential, inducing tumors in rodent models and disrupting key tumor suppressor pathways (13–15). As a nuclear-replicating DNA virus that relies entirely on host machinery for RNA processing, adenovirus has also been instrumental in advancing our understanding of RNA biology. Landmark discoveries made using AdV include mechanistic insights into RNA capping (16), RNA cleavage and polyadenylation (17), and the Nobel Prize-winning discovery of RNA splicing (18, 19). Through extensive use of alternative RNA processing, AdV produces nearly 100 unique RNA isoforms from a 36,000 base-pair viral genome that is only slightly larger than a single typical human gene. This remarkable information density is achieved by bidirectional transcription from both strands of the viral genome, combined with finely tuned regulation by RNA-binding proteins and RNA modifications (20–23). Understanding how such complex regulatory networks are encoded in a small genome is essential for precise genetic manipulation, as any modification of one gene can impact the regulation of a neighboring or antisense viral gene.

Numerous AdV reporters exist for both replication-competent and -incompetent models (24–28). The widespread use of well-established commercial systems such as Agilent’s AdEasy and Takara’s Adeno-X has led to a focus on reporters inserted into the E1 region, which is deleted in replication-incompetent vectors. These systems are largely restricted to studying early viral gene expression or require the use of complementing cell lines such as HEK-293. Some vector designs increase cargo capacity by deleting the E3 region, which encodes immunomodulatory proteins that are dispensable for replication in cell culture but important for immune evasion *in vivo* (29). Many E3 deletions have been imprecise, in some cases removing portions of the recently discovered antisense UXP transcript, and in most cases eliminating the polyadenylation signal that regulates late L4 transcripts (30, 31). A recently developed platform, “AdenoBuilder,” has further streamlined adenovirus reverse genetics (32). This system divides the Adenovirus serotype C5 (Ad5) genome into seven modular blocks, each maintained as a standard plasmid. These blocks can be linearized and recombined in a single reaction before transfection into human cells, enabling rapid, modular assembly of custom viral genomes (33). During the creation of this manuscript, an outstanding report was published that used AdenoBuilder to address many of the above concerns while dramatically expanding the toolbox of Ad5 reporters (34). In this publication, King et al. reported the use of multiple fluorescent viruses that faithfully recapitulate gene expression at both early and late times of infection. While the King et al. system utilized porcine teschovirus 2A (P2A) self-cleaving peptide systems to insert reporters in otherwise fully wild-type viruses, many of the recombinant viruses exhibited impaired replication kinetics compared to wild type, with reductions as large as 100-fold for more complex constructs. While all reported constructs remain modular within the AdenoBuilder system, we believe that there is further room for improvement.

Utilizing the combined power of AdenoBuilder reverse genetics and our own map of Ad5 transcription at unprecedented resolution (22), we have set out to build a new class of adenovirus reporter constructs. Our goal was to avoid the use of exogenous promoters or artificial splicing elements by harnessing the native regulatory architecture of the Ad5 genome to accurately reflect the timing and dynamics of viral gene expression. We successfully generated wild-type-like reporter viruses with replication kinetics nearly indistinguishable from unmodified virus, as well as replication-competent mutants capable of expressing multiple transgenes from a single, alternatively spliced transcriptional unit. Using these fluorescent systems, we established methodologies for rapid quantification of viral yield and real-time measurement of replication kinetics. This next generation of advanced adenovirus reporters enables precise study of viral gene regulation while also providing a robust and modular platform for future studies and high-throughput screening methods.

## RESULTS

### Update and design of AdenoBuilder reporter viruses

**Fig 1:**
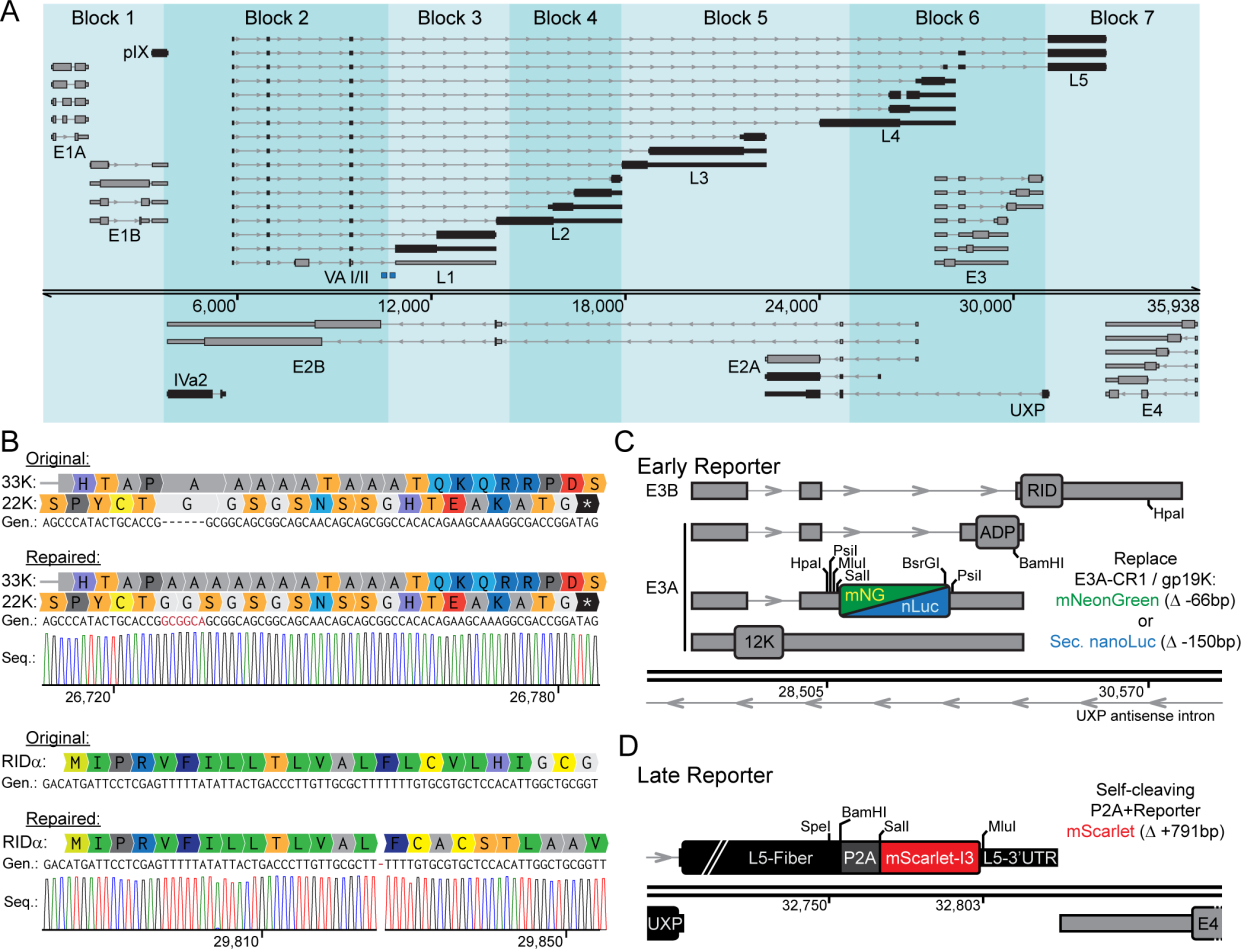
Construction of reporter viruses using a modified AdenoBuilder method. (**A**) Ad5 transcriptome map overlaid with AdenoBuilder plasmid blocks. Early genes are shown in gray, late genes are shown in black. (**B**) Schematic of the deletion and frame-shifting insertion in plasmids B6 and B6/7 of the Adenobuilder system. (**C**) Visual representation of the cloning method used to insert either mNeonGreen or secreted nanoLuciferase into the E3 early region. (**D**) Visual representation of the cloning method used to insert P2A-mScarlet into the Fiber late region.

Adenovirus contains numerous early (E) and late (L) transcription units that could be exploited to create reporters of viral gene expression (Fig. 1A). Importantly, noncoding regions within RNA introns contain regulatory information for the correct splicing of messenger RNAs, and both top- and bottom-strand RNA transcripts must be considered when designing a mutation or insertion that naturally affects both strands of DNA. Our group and others have recently used a combination of long-read and short-read RNA sequencing to accurately map start sites, cleavage sites, and long-range splicing patterns of all canonical Ad5 transcripts (20–22). When overlaid onto a map of the seven genomic blocks defined by the BstBI restriction enzyme cut sites from the AdenoBuilder system, the overlap of top-strand RNA, bottom-strand RNA, and noncoding elements is clearly seen.

When sequencing original AdenoBuilder system plasmids, we discovered five mutations that differed from the updated Ad5 reference genome. Three mutations involved single-nucleotide insertions or deletions that were located within the intergenic space between L1/L2, L3, and E4orf3/E4orf4, but were not predicted to have any effect on coding capacity or RNA processing. However, two In/Dels encoded for large truncations or frame-shifting mutations, both within Block 6 (Fig. 1B). These mutations were reported in the original AdenoBuilder publication (32), but we nevertheless endeavored to replace them with reference sequences. This included the re-insertion of a six nucleotide 5′-GCGGCA-3′ sequence within the reading frames of both L4-22K and L4-33K, as well as removal of a single T insertion that frame-shifted E3-RIDα. Subsequently, both mutations fixed in the single Block 6 plasmid were subcloned into the combined Block 6/7 plasmid. While neither mutation was likely to affect viral replication *in vitro*, we now possess the material to make an Ad5 virus that fully reflects the reference sequence.

We next set out to create a reporter construct for viral entry and the expression of early viral genes. While other groups have utilized the E1 region as the first expressed viral transcript, we instead utilized the E3 region, which is driven by an E1A-dependent early promoter (29). To minimize genomic disruption while maintaining genome size, we replaced the coding sequence of E3-CR1/gp19K with a reporter construct while preserving the upstream splicing signals, the downstream Adenovirus Death Protein (ADP) gene, and the E3A polyadenylation site (Fig. 1C). E3-gp19K is a membrane protein known to inhibit MHC class I presentation, whereas ADP plays a role in cell lysis and viral spread, justifying the deletion of gp19K and retention of ADP (35). The design also incorporates multiple restriction enzyme cut sites throughout the region to aid in future cloning using standard methodology. Two early reporter transgenes were constructed, containing either fluorescent mNeonGreen (mNG) or a secreted form of Nanoluciferase (Sec. nLuc) (36, 37).

To construct a reporter for late viral gene expression, we targeted the L5-Fiber region (Fig. 1D). L5 was selected because Ad5 encodes a single Fiber ORF, minimizing the risk of disrupting upstream or downstream RNA processing. As the most distal transcript within the Major Late Transcriptional Unit (MLTU), L5 is also particularly sensitive to changes in late-stage viral gene regulation, making it an ideal candidate for a late reporter (23). We inserted a self-cleaving P2A tag at the C-terminus of the Fiber ORF to avoid interference with the antisense UXP promoter located at the N-terminus. Importantly, the predicted cis-regulatory elements required for L5 polyadenylation overlap with the canonical ORF stop codon. To preserve these elements, we left this region intact and re-inserted the last 12 amino acids/36 nucleotides of the Fiber C-terminus as a codon-shuffled sequence upstream of the P2A tag to prevent premature polyadenylation. Downstream of the P2A cleavage site was inserted mScarlet-I3, a rapidly maturing version of the red fluorescent protein mScarlet3, which is spectrally orthogonal to the green fluorescent protein used in our early reporter (38). Because the early and late reporters are located in Blocks 6 and 7, respectively, they can be introduced individually or combined into a dual-color, replication-competent AdV.

### Rapidly assessing viral titer and infection dynamics with fluorescence-forming assays

**Fig 2:**
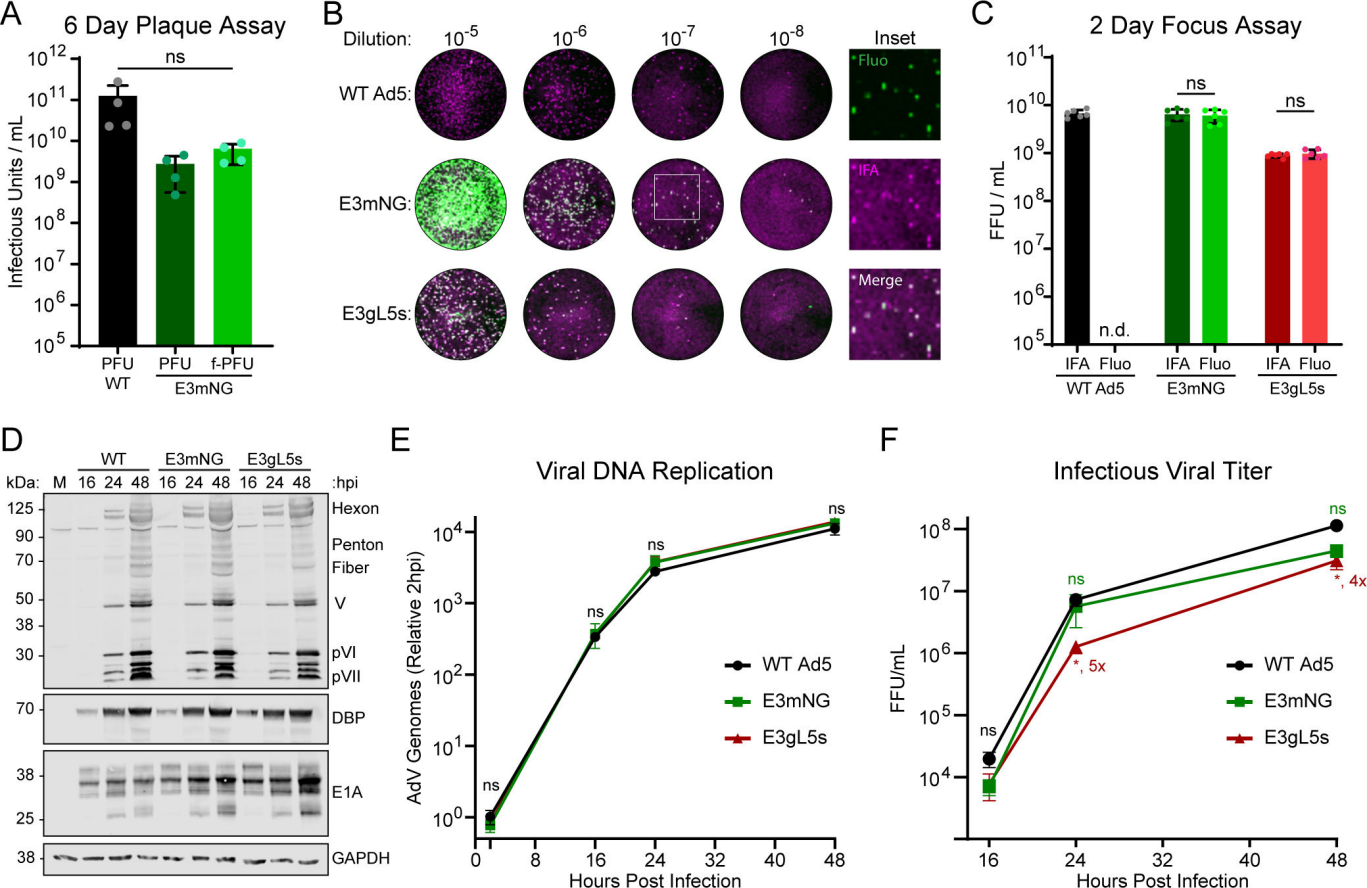
Reporter virus growth kinetics mimic that of wild-type AdV. (**A**) Viral titers measured in HEK-293 cells by conventional plaque assay (PFU) quantification or fluorescent plaque assay (fPFU) for E3mNG reporter virus compared with WT AdV. (**B**) In-cell western images of fluorescent reporters compared with WT AdV. Virus-encoded mNG expression (Fluo) overlaps with antibody staining for late viral proteins (IFA) at 48 hpi. (**C**) Titers of WT AdV and fluorescent reporter viruses measured by quantifying the number of cells stained with late viral proteins (IFA) or the number of cells expressing mNG green fluorescence (Fluo). (**D**) Representative western blot of A549 cells infected with either Mock (M), WT AdV, E3mNG, or E3gL5s for 16, 24, or 48 hours. Cell lysate was probed for Ad5 capsid proteins to assess late expression and DBP and E1A to assess early expression. GAPDH was used as a loading control. (**E**) qPCR analysis of viral genome replication in A549 cells. Cells were infected with either WT AdV, E3mNG, or E3gL5 and collected at either 2, 16, 24, or 48 hpi. Values were internally normalized to cellular Tubulin. (**F**) In-cell western quantification of infectious viral titer produced in A549 cells. Cells were infected with either WT AdV, E3mNG, or E3gL5s and collected at either 16, 24, or 48 hpi. The collected virus was then titered via in-cell western in A549 cells by measuring late viral protein signal. Statistics were determined by an unpaired two-tailed t test; *P < 0.05; ns, not significant; nd, not detected.

Infectious titer of AdV is typically determined by plaque-forming assay, in which a monolayer of cells is infected with serial dilutions of virus and overlaid with solidified agarose. The plaque-forming assay is time consuming (6–7 days prior to readout) and is sensitive to handling conditions. After a successful launch and amplification of wild-type Ad5 (WT), E3-driven early mNeonGreen reporter (E3mNG), and dual-color E3-green/L5-scarlet (E3gL5s), we titered our stocks by conventional plaque assay (Fig. 2A). Prior to staining, we observed green fluorescence marking individual plaques, and the number of fluorescent plaque-forming units per milliliter (fPFU/mL) was indistinguishable from the standard plaque-forming units per milliliter (PFU/mL). Thus, endogenously encoded fluorescence is amenable to quantifying infectious units in a sample of reporter virus.

We sought to further streamline the process of quantifying fluorescent viral titers by testing our infection system with the advanced LI-COR Odyssey M laser-based imaging platform. This system allows for simultaneous imaging of green fluorescent proteins with a 488 nm laser and near-infrared lasers for fluorescent antibody-based imaging. By adapting the Licor protocol for “in-cell westerns,” we infected A549 monolayers in standard 96-well plates with serial dilutions of wild-type Ad5 and successfully detected single-fluorescent foci using an anti-Ad5 capsid primary antibody and infrared secondary antibody (Fig. 2B). These foci decreased linearly with viral dose, and signal-to-noise was sufficient to exclude any signal in uninfected controls. Since 48 hours post-infection (hpi) represents a single round of Ad5 replication, we infer that each fluorescent focus represents a single infected cell, allowing this method to accurately quantify infectious particles in solution. Rather than plaque-forming units, this assay outputs fluorescence-forming units (FFU), and we distinguish this antibody-based approach from endogenous fluorescence as an Indirect Immunofluorescence Assay IFA-FFU assay.

To evaluate the compatibility of our reporter viruses with fluorescence-based titering, we infected A549 monolayers with either the early reporter virus (E3mNG) or the dual-reporter virus (E3gL5s) and performed IFA-FFU assays. Antibody-based staining detected infectious foci with similar efficiency across all three viruses. Importantly, imaging with the 488 nm laser also revealed strong mNeonGreen fluorescence from the early E3 reporter cassette in both reporter viruses, but not WT (Fig. 2B). At high viral dilutions, these signals appeared as distinct fluorescent foci that colocalized with antibody-detected foci (Fig. 2B, inset). We independently quantified infectious titers using either IFA-based FFU or mNeonGreen-based FFU for each virus and found no significant difference between the two methods (Fig. 2C). Furthermore, titers determined using the 2-day FFU assay closely matched those obtained from the conventional 6-day PFU assay (Fig. 2A and C). Thus, fluorescence-based plate scanning allows for a streamlined approach for quantifying infectious titers of both wild-type and reporter-encoding AdV.

### Fluorescent reporter virus kinetics mimic that of wild-type AdV

Our fluorescent reporter viruses were designed to closely mimic wild-type adenovirus replication kinetics while enabling rapid analysis of the viral life cycle. To assess their ability to produce viral proteins over time, A549 cells were infected with a multiplicity of infection (MOI) of 10 FFU/cell with wild-type Ad5, the early reporter (E3mNG), or the dual-color reporter (E3gL5s). Samples were collected at 16, 24, and 48 hpi and analyzed by western blot for expression of early (E1A, DBP) and late structural proteins (Fig. 2D). All viruses showed comparable protein expression profiles, with early proteins detectable by 16 hpi and late proteins peaking at 48 hpi. As a measure of early infection, we assessed viral DNA accumulation by quantitative PCR at the same time points (Fig. 2E). Viral genome replication was superimposable across all viruses, with no statistically significant differences between wild-type Ad5 and either reporter construct. Finally, to examine the kinetics of infectious virion production, we performed IFA-FFU assays using total cell lysates harvested at 16, 24, and 48 hpi (Fig. 2F). E3mNG produced slightly fewer infectious particles compared to wild type, though differences were not statistically significant. The dual-color E3gL5s virus showed a modest fourfold to fivefold reduction in titer at 24 and 48 hpi, but followed the same overall replication kinetics as wild-type Ad5. Thus, both of our early- or late-stage reporter viruses faithfully recapitulate early and late stages of viral replication with kinetics that closely mimic the wild-type virus.

### Fluorescent imaging of reporter viruses in living cells recapitulates viral infection stages

**Fig 3:**
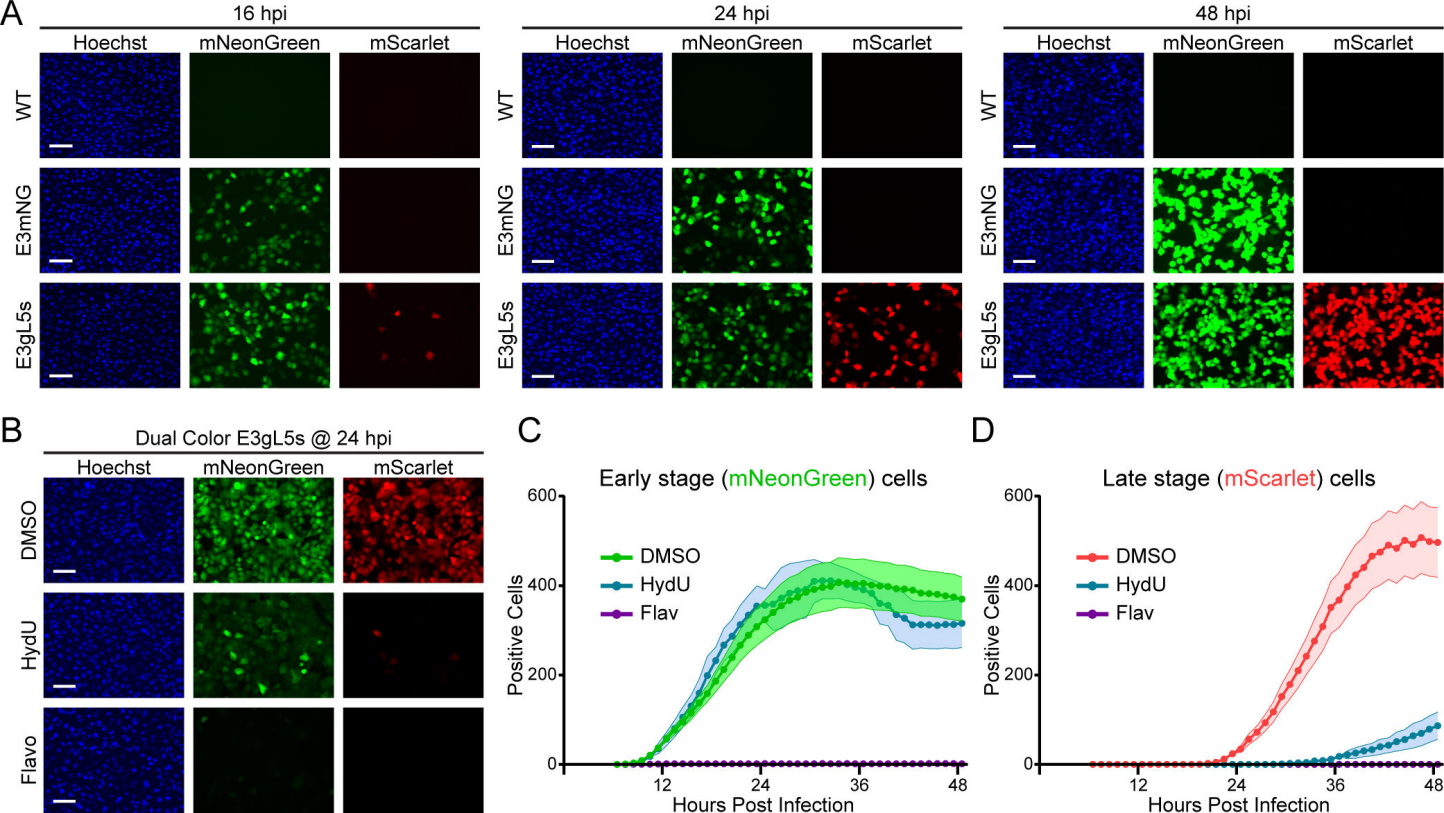
Imaging of fluorescent reporters recapitulates the viral replication cycle of AdV. (**A**) Fluorescence microscopy analysis of A549 cells infected with either WT AdV, E3mNG, or E3gL5s at 16, 24, or 48 hpi. Early reporter expresses mNG (green) and late reporter expresses mScarlet (red). (**B**) Fluorescence microscopy analysis of A549 cells infected with E3gL5s, treated with DMSO, Hydroxyurea (1 mM), or Flavopiridol (300 nM), and imaged at 24 hpi. (**C**) CellCyte analysis of early reporter green fluorescent positive A549 cells following E3gL5s infection for 48 hours after treatment with DMSO (green), Hydroxyurea (HydU, blue), or Flavopiridol (Flav, purple) at 6 hpi. The shaded area represents the SEM of six replicates. (**D**) CellCyte analysis of late reporter red fluorescent A549 cells following E3gL5s infection for 48 hours after treatment with DMSO (red), Hydroxyurea (HydU, blue), or Flavopiridol (Flav, purple) at 6 hpi. The shaded area represents the SEM of six replicates. For all imaging, the white scale bar represents 100 µm.

Fluorescent viral reporters enable imaging of living cells and a window into the dynamics of infection in real time. To evaluate the temporal kinetics of our early and late gene reporters, we infected A549 cells at an MOI of 10 FFU/cell with non-fluorescent wild-type Ad5, early reporter E3mNG, or dual-reporter virus E3gL5s. Infected wells were sequentially imaged at 16, 24, and 48 hpi for green fluorescence, red fluorescence, and nuclear DNA staining with Hoechst (Fig. 3A). As expected, cells infected with wild-type Ad5 exhibited no detectable fluorescence at any time point. The early reporter E3mNG showed green fluorescence in nearly all cells as early as 16 hpi, with fluorescence intensity increasing modestly over time. The dual-reporter E3gL5s displayed a similar pattern of green fluorescence throughout the time course, but at 16 hpi only a small number of cells exhibited red fluorescence. This is consistent with the known kinetics of AdV late gene expression (22, 39, 40). As time post-infection progressed, more cells displayed red fluorescence, and the individual cell brightness increased, until nearly all cells were bright red by 48 hpi. These data demonstrate that our early reporter virus accurately measures early gene expression, while the dual reporter’s late gene expression cassette is tied to the replication kinetics of AdV.

We next tested whether our dual-reporter virus could sensitively respond to cellular perturbations known to differentially affect distinct stages of adenovirus replication. Because late gene expression is dependent on successful viral DNA replication, we treated A549 cells with hydroxyurea 5 hours post-infection to deplete cellular nucleotide pools and induce replication stress. At 24 hpi, DMSO-treated control cells expressed both green and red fluorescence, reflecting active early and late gene expression. By contrast, hydroxyurea-treated cells showed only green fluorescence from the early reporter, consistent with inhibition of DNA replication and loss of late gene expression (Fig. 3B). To assess the requirement for *de novo* transcription, we treated infected cells with the CDK9 inhibitor flavopiridol after infection to block RNA polymerase II-mediated transcription. Under these conditions, neither green nor red fluorescence was detected, indicating a complete block of reporter gene expression. Thus, our dual-reporter virus confirms that early reporter expression depends on active transcription shortly after infection, while late reporter expression additionally requires viral genome replication. Together, this system faithfully replicates the early and late stages of AdV infection.

To capture the dynamics of early and late gene expression with high temporal resolution, we used a CellCyte automated live-cell imaging system to image infected A549 cells every hour for 48 hours. Cells were infected with the dual-reporter E3gL5s in the presence of DMSO, hydroxyurea, or flavopiridol and monitored for green and red fluorescence over time. In DMSO-treated controls, green fluorescence appeared within the first 12 hours post-infection and progressively increased, followed by the emergence of late-gene red fluorescence around 24 hours (Fig. 3C and D). In cells treated with hydroxyurea, green fluorescence developed with similar timing and levels to DMSO controls, confirming that early gene expression proceeds independently of viral DNA replication (Fig. 3C). However, red fluorescence was substantially abrogated, indicating that late gene expression was blocked (Fig. 3D). Likewise, flavopiridol treatment suppressed both green and red fluorescence, consistent with the requirement for active RNA polymerase II transcription to initiate infection. These real-time imaging data reinforce the conclusion that our dual-reporter virus accurately separates the early transcriptional activation phase from the replication-dependent late gene expression program.

### AdV luciferase reporter enables rapid and quantitative measurements of viral replication

**Fig 4:**
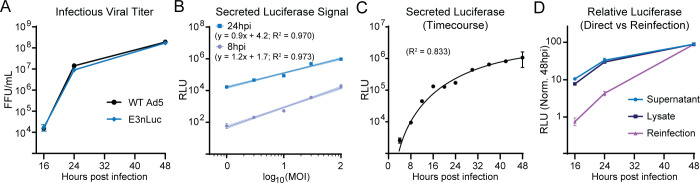
Optimized luminescence-based assay to assess the growth kinetics of secreted luciferase reporter virus. (**A**) IFA-FFU quantification of E3nLuc infection compared to that of wild-type Ad5. Cells were infected with either WT AdV or E3nLuc and collected at 16, 24, or 48 hpi. The collected virus was titered by IFA for late viral protein signal. (**B**) Comparison of luciferase expression in A549 cells infected with E3nLuc, in which supernatant was collected at either 8 or 24 hpi. Cells were infected at MOIs of 1, 3, 10, 30, and 100. Linear regression with shaded 95% confidence intervals, line formula, and R^2^ value is shown. (**C**) Analysis of secreted luciferase from E3nLuc infection over time. Supernatant was collected every 4 hours until 48 hpi. Best-fit line of nonlinear regression with an R^2^ value is shown. (**D**) Comparison of direct vs. reinfection secreted luciferase assays. Cells were infected with E3nLuc and collected at either 16, 24, or 48 hpi. Samples were then immediately read for Luciferase activity (Supernatant), freeze-thawed three times, and then read for luciferase activity (Lysate), or equal volumes were used to reinfect naïve A549 cells for 6 hours at a 1:10 dilution (Reinfection) before being collected and read for luciferase enzyme activity. All samples were normalized to the average of their respective 48 hpi sample set to 100.

While fluorescent proteins are invaluable for isolating living cells, they are not optimal for quantitatively measuring absolute reporter gene expression. By contrast, luciferase enzymes provide a robust, highly sensitive bioluminescent signal with a wide linear dynamic range spanning several orders of magnitude. Luciferase assays are well-suited for high-throughput, plate-based formats; however, conventional assays typically require cell lysis, precluding longitudinal analysis from the same sample. To overcome this limitation, we employed Promega’s secreted Nanoluciferase (nLuc) fused to an IL-6 secretion signal, allowing quantification of reporter activity directly from culture supernatants over time (37). We engineered an adenovirus reporter expressing secreted nLuc (E3nLuc) under control of the same E3 promoter used in our early fluorescent reporter construct (Fig. 1C). To confirm that the insertion of nLuc did not impair replication, we compared infectious titers of WT Ad5 and E3nLuc at multiple time points post-infection and observed no significant differences by IFA-FFU assay (Fig. 4A). To evaluate the dynamic range of nLuc output, we infected cells with increasing MOIs and measured luciferase activity in supernatants at 8 and 24 hpi (Fig. 4B). Relative luciferase light units (RLU) scaled linearly with MOI at both time points (best-fit slopes of 1.2x and 0.9x at 8 and 24 hpi, respectively), and luciferase activity at 24 hpi was approximately 100-fold higher than at 8 hpi, consistent with increased gene expression and viral replication. To assess the system’s ability to support longitudinal monitoring, we performed a 48 hour time course with supernatant aliquots collected from the same infected wells every four hours (Fig. 4C). Luciferase activity was detectable as early as 4 hpi, increased steadily, and peaked around 36 hpi before plateauing. These results demonstrate that secreted nLuc, driven by the endogenous E3 promoter, provides a sensitive, quantitative, and non-destructive method for real-time monitoring of adenoviral gene expression kinetics.

While early promoter-driven reporters are useful for tracking initial stages of infection, their signal may not scale proportionally with late-stage outcomes such as infectious virion production. To test this, we infected A549 cells with E3nLuc and harvested samples at 16, 24, and 48 hpi. For each sample, we collected (i) an aliquot of supernatant alone, (ii) the remaining cells plus supernatant after scraping and lysis by three freeze-thaw cycles and (iii) the clarified lysate from above used to reinfect naïve A549 cells. Subsequently, all conditions were assayed for luciferase activity and reported as relative light units (RLU), normalized to the 48 hpi timepoint for each condition (Fig. 4D). RLU levels from both supernatant and total lysate of the original infection increased approximately 10-fold from 16 to 48 hpi, consistent with the accumulation of secreted luciferase. Upon reinfection, however, there was a much steeper increase of nearly 10-fold between 16 and 24 hpi, and a 100-fold increase from 16 to 48 hpi. These data demonstrate that reinfection-based readouts provide greater dynamic range for measuring late-stage events, including successful virion production. Importantly, this approach effectively converts an early-reporter virus into a surrogate for late-stage output. Researchers can thus choose between the simplicity and speed of direct supernatant sampling versus the enhanced sensitivity and dynamic range of a reinfection-based assay.

### Mutant viruses can be repurposed for reporter gene expression

**Fig 5:**
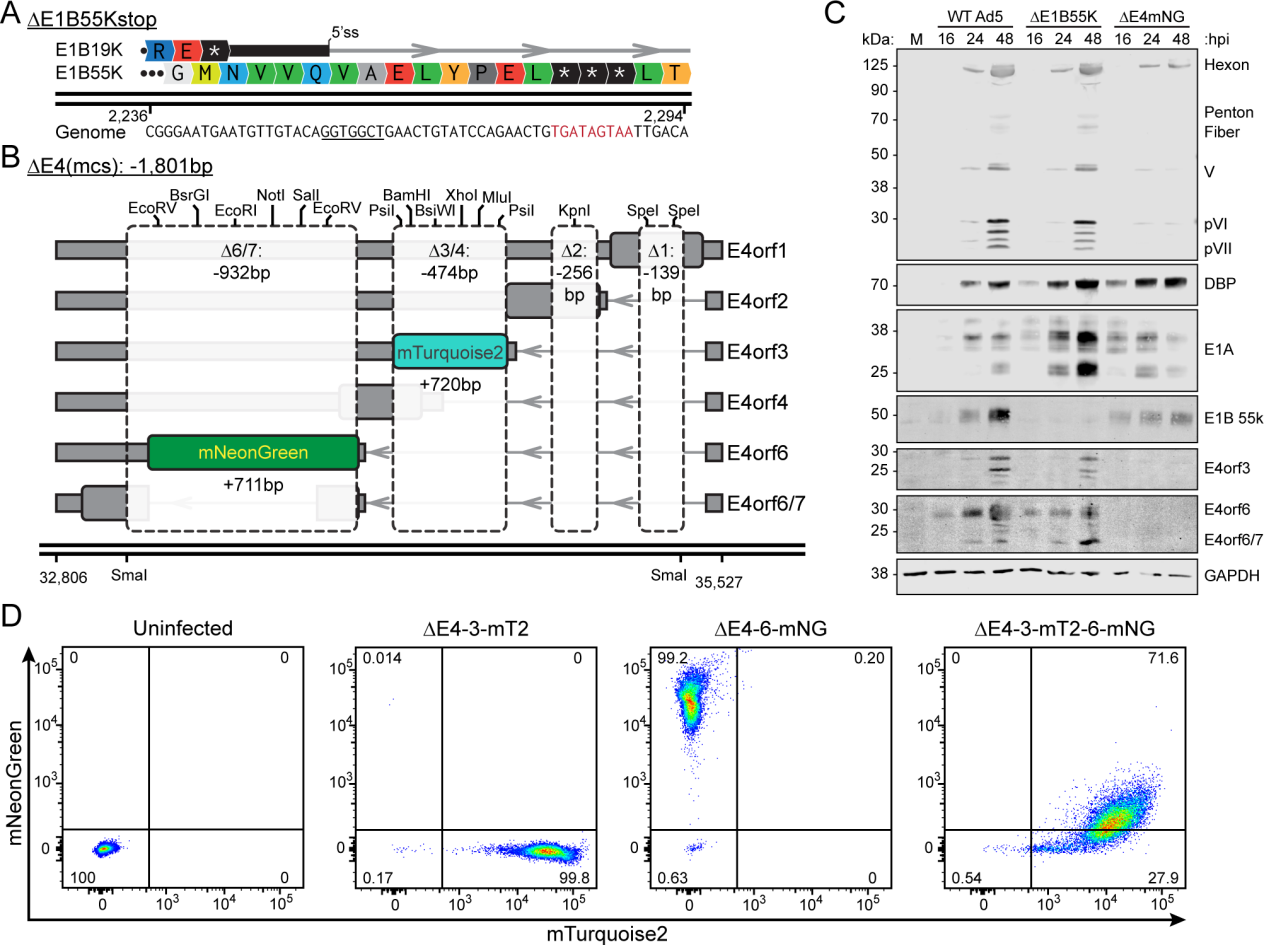
Construction of fluorescent-tagged mutant viruses with endogenous processing. (**A**) Schematic of the construction of △E1B55K. E1B19K 5’ splice site is underlined. (**B**) Schematic of the construction of △E4-6-mNG, △E4-3-mT2, and △E4-3-mT2-6-mNG. The mNeonGreen tag was inserted in orf6 of △E4(mcs), while the mTurquoise2 tag was inserted in orf3. (**C**) Representative western blot of HeLa cells infected with either Mock (M), WT AdV (MOI 10), △E1B55k (MOI 10), or △E4-6-mNG (MOI 100) for 16, 24, or 48 hours. Cell lysate was probed for AdV5 capsid proteins to assess late expression and DBP and E1A to assess early expression. Cell lysate was also probed for E1B55k, E4orf3, and E4orf6 to confirm knockouts. GAPDH was used as a loading control. (**D**) Flow cytometric analysis of HeLa cells uninfected or infected with △E4-3-mT2, △E4-6-mNG, or △E4-3-mT2-6-mNG (MOI 100) and analyzed for mNeonGreen and mTurquoise2 expression at 48 hpi. The percentage of positive cells for each fluorophore is present in each gated quadrant.

Previous generations of adenovirus recombinants were often created by subcloning restriction fragments into shuttle vectors or exploiting rare, single-cut restriction sites within the viral genome to facilitate recombination. These approaches frequently resulted in deletions made at convenient, but not necessarily optimal, locations. By contrast, the AdenoBuilder system enables rapid and seamless introduction of mutations or insertions at nearly any position in the genome via PCR-based strategies. As proof of principle, we designed a deletion mutant of the viral E1B-55K protein by inserting three in-frame stop codons in a manner that preserves upstream splicing of E1B-19K (Fig. 5A). E1B-55K is a multifunctional viral protein that contributes to an AdV-mediated ubiquitin ligase complex targeting host factors involved in DNA damage responses and RNA processing. Mutations in the E1A and E1B regions have been well characterized due to their ability to be easily *trans*-complemented by complementing HEK-293 cells.

AdV contains another modular region on the right end of its genome that encodes the bottom-strand E4 transcriptional unit. This unit is driven by a single promoter and polyadenylation site, yet through alternative splicing generates six distinct ORFs. The resulting E4 proteins contribute to evasion of host antiviral defenses, often in coordination with E1B-55K, and can be functionally complemented in W162 Vero cells (41). Historically, mutations in this region have focused on single E4 ORFs or involved large deletions that disrupted multiple ORFs along with their associated regulatory sequences. To exploit the extensive alternative splicing of this region, we engineered a synthetic deletion within the AdenoBuilder Block 7 plasmid creating a △E4 (multiple cloning site) (Fig. 5B). While this deletion introduces large, frame-shifting mutations across all six E4 ORFs, it preserves the essential RNA elements including endogenous E4 promoter, exon 1 5′ splice donor, and all downstream 3′ splice acceptors and polyadenylation site. Into this engineered locus, we inserted transgenes at specific splice acceptor sites to generate three unique viruses: cyan fluorescent protein mTurquoise2 driven by the E4orf3 splice site (△E4-3-mT2), green fluorescent mNeonGreen driven by the E4orf6 splice site (△E4-6-mNG), or a combination containing both mTurquoise and mNeonGreen from their respective splice acceptors (△E4-3-mT2-6-mNG). This strategy leverages the native RNA processing architecture of the viral genome to create multicistronic reporter constructs that are fundamentally distinct from other conventional approaches that rely on internal ribosome entry sequences or 2A self-cleaving peptides.

Western blot analysis in HeLa cells confirmed successful generation of both △E1B55K and △E4 viruses, with specific loss of the E1B-55K protein and all E4 gene products, along with the expected reduction in accumulation of late viral capsid proteins (Fig. 5C). To assess reporter expression, HeLa cells were infected with one of the three △E4 viral reporters and analyzed by flow cytometry at 48 hpi (Fig. 5D). In all cases, greater than 99% of cells were positive for fluorescence, and the spectrally similar mTurquoise2 and mNeonGreen reporters were readily resolved in single-color infections. Notably, infection with the dual-reporter △E4-3-mT2-6-mNG virus resulted in ~75% of cells co-expressing both fluorophores. On a per-cell basis, mNeonGreen driven by the E4orf6 splice site was dimmer than in cells infected with the single-color ∆E4-6-mNG virus, potentially reflecting competition between splice acceptor sites. In the natural context of wild-type infection, E4orf3 mRNA accumulates earlier than E4orf6 mRNA, suggesting that our exogenous reporter system retains aspects of native splicing regulation (22, 39, 40).

### Single-vector system for doxycycline-inducible transgene expression

Replication-incompetent AdV vectors are widely used for their ability to transduce non-dividing or hard-to-transfect cells. While providing relatively high cargo capacity compared to other viral vectors, this space becomes limiting when both coding sequences and inducible regulation are required. A commonly used system for inducible expression is the tetracycline transactivation system, which requires trans-encoded reverse tetracycline transactivator (rtTA) to drive expression from tetracycline-responsive promoters in the presence of doxycycline (Dox). Current best practices for Dox-inducible AdV vectors require prior engineering of the target cell to express rtTA, encoding tandem or diverging promoter units inside the E1 deletion driving both rtTA and the gene of interest, or co-infection of the same cell with two separate recombinant AdV expressing rtTA and the regulated gene of interest on separate vectors (42, 43). Leveraging the modularity of the AdenoBuilder platform, we sought to determine whether both rtTA and the inducible transgene could be encoded in distinct regions of a single recombinant AdV genome.

**Fig 6:**
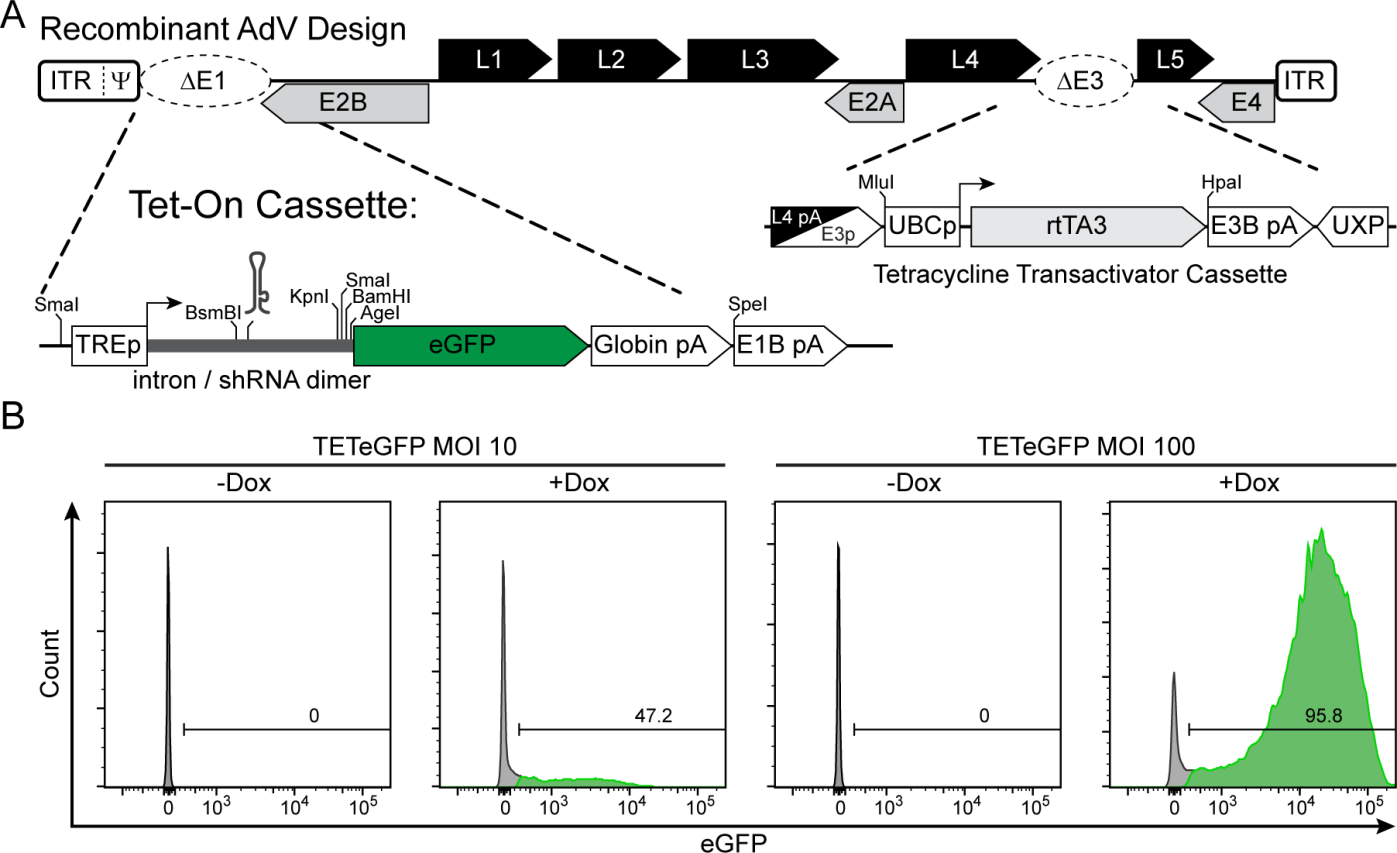
Recombinant AdV vector system for tetracycline-inducible transgene expression. (**A**) Schematic of recombinant AdV design with E1 Tet-On cassette and E3 reverse Tetracycline Transactivator (rtTA3) cassette. (**B**) Flow cytometric analysis of HeLa cells infected with TETeGFP at either MOI 10 or 100. Doxycycline (1 µg/mL) (+Dox) or DMSO (−Dox) treatment was added at 2 hpi, and cells were analyzed for eGFP expression at 48 hpi.

We adapted a previously described single-vector doxycycline-inducible system by integrating its two components into separate regions of the AdV genome using AdenoBuilder (44). Using our modified Block 6 E3 region reporter construct, we deleted the entire E3 region while sparing upstream L4 polyadenylation sites and downstream UXP reading frame. In its place, we inserted a ubiquitous cellular promoter driving the rtTA3 ORF that terminates at the endogenous E3B polyadenylation site (Fig. 6A). Separately, in Block 1, we replaced the E1A/E1B region with a tetracycline-responsive element (TREp) driving enhanced GFP (eGFP), preceded by a chimeric intron for improved expression. The intron was engineered to include a pre-miR-155 backbone, allowing for modular insertion of shRNA sequences (44). These modified blocks were assembled into a single replication-incompetent AdV. Vector titration was performed using a modified IFA-FFU assay detecting viral capsid proteins in replication-complementing HEK-293 cells. Furthermore, eGFP expression could be seen in a matching FFU assay in HEK-293s in the presence of doxycycline. This method validated the function of both regulatory modules within a single vector.

To assess the stringency of doxycycline-dependent regulation, HeLa cells were transduced with either 10 or 100 FFU/cell of the inducible AdV and cultured with or without doxycycline for 48 hours. At an MOI of 10, approximately 50% of cells expressed strong eGFP fluorescence in the presence of doxycycline, as measured by flow cytometry (Fig. 6B). At an MOI of 100, over 95% of cells were successfully transduced and showed high levels of eGFP expression with doxycycline treatment. Notably, no eGFP-positive cells were detected in the absence of doxycycline at either MOI. These results demonstrate that regulated gene expression from this single-vector system is both robust and strictly dependent on doxycycline.

## DISCUSSION

### Utilizing existing viral regulatory elements for reporter design

In this study, we leveraged the modular AdenoBuilder system and a high-resolution map of Ad5 transcription to develop a next-generation toolkit of adenoviral reporter vectors. These include replication-competent viruses with fluorescent reporters integrated into native early and late transcription units, enabling precise, real-time visualization of viral gene expression. We demonstrated that these constructs preserve wild-type replication kinetics and can be quantified using streamlined, fluorescence-based infectious unit assays. We further incorporated secreted Nanoluciferase reporters for non-destructive, longitudinal measurement of viral activity and developed single-vector systems for tightly regulated, doxycycline-inducible expression in replication-incompetent backbones. Together, these tools establish a flexible and quantitative platform for monitoring adenoviral infection dynamics and manipulating gene expression within native regulatory contexts.

### Utility of real-time and non-invasive assays

Real-time, non-invasive assays are invaluable tools for dissecting the complex dynamics of viral replication with minimal perturbation to the biological system. Single- and dual-color fluorescent reporters allowed for the use of CellCyte, or other high-content imaging systems, to enable continuous imaging every hour over a 48 hour period, providing unprecedented insight into the timing and coordination of early and late gene expression in individual infected cells. Furthermore, our E3mNG-based FFU assay uses endogenous green fluorescence from an early viral promoter-driven reporter, allowing direct quantification of infectious units. Coupled with an antibody-based IFA-FFU assay, we can also determine the infectious titer of unlabeled viruses in an optimized protocol that reduces assay time from days to hours. The secreted Nanoluciferase reporter offers a robust, quantitative, and non-destructive longitudinal assay to monitor viral gene expression over time from culture supernatants. This approach allows kinetic studies of viral replication or antiviral interventions within the same sample, eliminating variability introduced by destructive sampling and enabling time-course experiments with minimal labor.

### Insights into viral gene regulation and kinetics

Our reporter viruses have proved useful for studying the intricate relationship between viral DNA replication and late gene activation. While it was well established that hydroxyurea impairs viral DNA replication (45, 46), the dynamic range of this system will allow for the study of additional compounds that inhibit DNA replication or late-gene RNA processing. These findings reinforce the fundamental role of viral DNA replication as a critical switch that licenses the expression of structural genes necessary for virion assembly. Similarly, our E4-region reporter system based on endogenous RNA processing presents new opportunities for exploring how RNA biology is regulated during infection (47). By exploiting native splice sites rather than relying on artificial 2A or IRES elements, this system provides a physiologically relevant model for studying post-transcriptional control of multicistronic transcripts. This includes how adenoviral E4 proteins regulate cellular splicing factors to regulate their own transcription (48, 49). Such studies would enhance our understanding of how adenoviruses co-opt host RNA machinery and could reveal new vulnerabilities for targeting the viral lifecycle.

### Applications and future studies

An obvious follow-up to this work is to leverage the utility and rapid readout of these AdV reporters for diverse platforms of high-throughput screening. In particular, the secreted luciferase system offers a highly sensitive, non-destructive, and quantitative readout that can be sampled repeatedly from the same well, making it ideal for longitudinal drug screening in 96- or 384-well formats. Because the luciferase signal scales with both multiplicity of infection and time post-infection, it enables precise dose-response measurements and kinetic profiling of antiviral compounds targeting early stages of the viral replication cycle. Alternatively, the dual-color fluorescent virus allows for distinguishing between viral entry, early-stage gene expression, and true late processes. Either modality can be applied to unbiased plate-based screening platforms. Existing small-molecule libraries or siRNA knockdown panels can be used to identify host pathways or druggable targets that modulate infection. Fluorescence-Activated Cell Sorting (FACS) can be coupled with pooled CRISPR screens to identify host genes that selectively control discrete stages of the adenovirus lifecycle. Many such drug and CRISPR screens have been done for AdV (50–53), or other DNA viruses (54–60), yielding impressive insight into viral biology.

The modularity of the AdenoBuilder system enables rapid and flexible incorporation of diverse reporter cassettes into virtually any recombinant adenovirus background, facilitating the study of specific viral gene functions in the context of infection. This theoretically extends to mixing and matching various early- or late-gene reporters from our study or others (34). For example, early gene expression can be tracked using the King et al. Block 1 E1A reporter or our Block 6 E3-driven reporters, while late gene expression can be monitored using their Block 4 L2-V construct or our Block 7 L5-Fiber reporters. Subsequently, these combinations can be introduced into recombinant virus backgrounds carrying targeted mutations in genes of interest, such as deletions in E1B-55K, E4 proteins, or late assembly proteins, to directly assess how those mutations impact different stages of the viral lifecycle. This design allows for side-by-side comparisons of viral gene regulation, genome replication, and particle production, with rapidly produced isogenic viruses.

In summary, these novel adenoviral reporter systems provide a powerful and modular platform for interrogating viral gene regulation, tracking replication in real time, and accelerating high-throughput discovery. By preserving authentic kinetics of infection through endogenous viral regulatory architecture, these tools offer new experimental precision for studying adenovirus and vector biology.

## MATERIALS AND METHODS

### Cell culture

Cell lines were grown in Dulbecco’s modified Eagle medium (DMEM; Corning, 10-013-CV) supplemented with 10% vol/vol fetal bovine serum (R&D Systems, S11550) and 1% vol/vol pen-strep (Corning, 30-002-CI) at 37°C and 5% CO2. All cell lines tested negatively for mycoplasma contamination using the MycoAlert Mycoplasma Detection Kit (Lonza, LT07-418). AD-293 (Agilent, 240085), A549 (ATCC, CCL-185), and HeLa cells (ATCC, CCL-2) were obtained commercially. Vero and W162 cells (E4 complemented Vero cells) were a generous gift from M. Weitzman.

### Cloning plasmids

Molecular cloning of existing AdenoBuilder plasmids (Addgene Kit #1000000176) was completed using various means. Unless stated otherwise, all PCR was performed with Q5 High-Fidelity 2X Master Mix (NEB, M0492), site-directed mutagenesis performed with KLD Enzyme Mix (NEB, M0554), and cloning with 2× NEBuilder HiFi DNA Assembly Master Mix (NEB, E2621L), all according to the manufacturer’s instructions. PCR primers and linear gBlocks were purchased from IDT. All DNA oligonucleotide sequences are found in Table S1.

To create the E3 reporter construct, plasmid B6 was linearized by PCR, and a gBlock containing mNeonGreen and surrounding restriction sites was inserted by NEBuilder HiFi reaction. The resulting plasmid was digested with MluI and BsrGI, and a secreted nLuc gBlock was inserted by NEBuilder HiFi reaction to create the E3nLuc plasmid. To create the L5 reporter construct, plasmid B7 was linearized by PCR, and a gBlock containing P2A-mScarletI3 and surrounding restriction sites was inserted by NEBuilder HiFi reaction. To create the △E4(mcs), plasmid B7 was digested with SmaI, and the entire multiple cloning site fragment was inserted by NEBuilder HiFi reaction. A SalI/BsrGI fragment containing mNeonGreen was digested from E3mNG and ligated into compatible SalI/BsrGI sites within the digested △E4(mcs) plasmid to create △E4-6-mNG. △E4(mcs) or △E4-6-mNG was digested with MluI/BamHI, and a gblock containing mTurquoise2 was inserted into the region controlled by E4orf3 splicing to generate both △E4-3-mT2 and △E4-3-mT2-6-mNG, respectively. The creation of E1B55Kstop plasmid and the repair of the 22K/33K and RIDα regions of B6 were performed by site-directed mutagenesis using Q5 PCR and KLD mix to ligate the resulting linear product.

The tetracycline-responsive eGFP system was modified from plasmid BB726 from Khandelia et al. (44). Using PCR primers with homology arms, the plasmid was amplified into two functional products containing either the UBC promoter-rtTA3 element or the TRE promoter-eGFP-Globin polyadenylation element. Plasmid B6-E3mNG was digested with HpaI, and the UBC-rtTA3 PCR product was inserted by NEBuilder HiFi reaction to create △E3A/B-UBC-rtTA3. Plasmid B1-deltaE1-mcs was digested with SmaI/SalI, and the TREp-eGFP PCR product was inserted by NEBuilder HiFi reaction to create △E1-TETeGFP. Successful construction of all constructs was confirmed by PlasmidSaurus whole-plasmid sequencing.

### Construction of viruses

Recombinant adenoviruses were created using a modified AdenoBuilder protocol (33). 500 fmol of each plasmid block was mixed together, digested with BstBI (NEB, R0519S), and incubated at 65°C for 1 hour. Plasmid DNA was then precipitated using a mixture of Ammonium Acetate (Fisher Chemical, A639-500) and ethanol (Decon Laboratories, Inc., 3916EA) at −20°C overnight. Isolated DNA was washed with 70% ethanol, dried, and resuspended in 10 μL of UltraPure water (Invitrogen, 10977023). The plasmid blocks were ligated together by adding an equal volume of 2X NEBuilder HiFi DNA Assembly Master Mix (NEB, E2621L) to the purified DNA and incubating at 45°C for 1 hour. 5 μL of assembled DNA (approximately 8 µg of DNA) was then used for the next steps. Viruses were either electroporated or transfected into 2 million cells, which were spread between two wells of a six-well plate for initial viral infection to occur. Unless otherwise specified, viruses were simply electroporated into AD-293 cells using the Lonza Walkersville SF Cell Line 4D-Nucleofector X Kit L (V4XC-2024) and the CM130 nucleofection protocol. The △E4mNG virus was transfected into W162 cells using 2:1 Xtreme Gene transfection reagent (Roche, 6366236001). The △E4-3-mT2 and △E4-3-mT2-6-mNG viruses AdenoBuilder transfection was spiked with 1 µg/well of pHelper (61) plasmid (NovoPro, V005569), a plasmid containing the full AdV E4 region, upon electroporation in AD-293 cells, and once again transfected with pHelper 3 days post-electroporation for a successful initial infection. The three △E4 viruses were then expanded in W162 E4 complementing cells, while all other viruses were expanded in AD-293 cells. The wild type, E3mNG, E3nLuc, E3gL5s, and △E4mNG viruses were purified using cesium chloride density centrifugation following expansion. All other viruses were freeze-thawed, cleared, and stored as crude preps. All viruses were titered via in-cell western and/or plaque assay.

### PFU titering

Plaque assay titering was performed in AD-293 cells. Cells were infected with wild-type Ad5 or E3mNG in serial dilutions from 10^−9^ to 10^−6^. After 2 hours in infectious 2% FBS vol/vol DMEM media supplemented with 1% vol/vol pen-strep, wells were aspirated and overlayed with plaque media. Plaque media was made from 14 mL of 2% DMEM plus 1.5 mL of 4% SeaPlaque (Lonza, 50101) in water. After the overlay was applied, the infection was left to progress for 5–7 days. Once plaques were clearly visible, cells were fixed with 10% trichloroacetic acid (TCA) (Millipore Sigma, T6399−4 × 100G) in 1× phosphate-buffered saline (PBS; Corning, 21-031-CV) for 30 minutes. Following fixation, agar and TCA were aspirated from wells, and cells were stained with 1% Crystal Violet (Millipore Sigma, C6158-50G) in 50% Ethanol for 30 minutes. Finally, plates were rinsed, and plaques were manually counted.

### FFU titering

FFU-based assays were performed in black 96-well plates (Greiner, 655090). Infectious media made up in 2% FBS vol/vol DMEM was supplemented with 1% vol/vol pen-strep, which was serially diluted and added to cells. The dilutions performed were typically 10^−9^ to 10^−5^, but they varied to ensure individual fluorescent and/or antibody-stained cells could be observed. At 2 hpi, infectious media was aspirated off cells; they were washed with 1× PBS (Corning, 21-031-CV), then topped with 10% vol/vol DMEM supplemented with 1% vol/vol pen-strep. At 48 hpi, plates were either read on the LiCor Odyssey M if viruses contained fluorescent tags, or an in-cell western (ICW) was performed. To perform an ICW, plates were first fixed with 4% paraformaldehyde (PFA) (Thermo Scientific, J19943K2) for 15 minutes and then rinsed with 1X PBS. Cells were then permeabilized with 0.5% Triton-X (Thermo Scientific, A16046AP) for 10 minutes before blocking with 3% Bovine Serum Albumin (BSA) (Fisher BioReagents, BP1600-1) plus 1× Sodium Azide (Millipore Sigma, S2002-500G) in PBS. Cells were probed for Anti-Ad5 Capsid at 1:5000 (Abcam, ab6982) or E1A at 1:2,000 (BD Biosciences, 554155) overnight at 4°C in 3% BSA. Anti-rabbit (LiCor, 926-68171) or anti-mouse (LiCor, 827-08364) secondary antibodies at 1:10,000 in 1× PBS (Fisher BioReagents, BP3991) were applied to the cells for two hours, after which the plates were scanned on a LiCor Odyssey M.

### Western blotting

A549 or HeLa cells were infected with the virus at an MOI of 10, with the exception of △E4 viruses, which were infected at an MOI of 100. Wells were infected in triplicate and lysate was collected at 16, 24, and 48 hours post-infection. At each time point, wells were aspirated, rinsed with 1× PBS (Corning, 21-031-CV), and lysed with 200 μL of lysis buffer (1:4 NuPAGE LDS Sample Buffer to Ultrapure Water, supplemented with 1 mM DTT). Lysed samples were boiled at 95°C for 15 minutes, cooled, then vortexed and spun down before loading. Proteins were separated in precast NuPAGE polyacrylamide gels, or self-cast using the Invitrogen SureCast Gel Handcast System, and transferred onto nitrocellulose membranes (Bio-Rad, 1620115). Membranes were blocked with 5% non-fat milk (Lab Scientific, M0841) plus 1× Sodium Azide (Millipore Sigma, S2002-500G) and then rocked in primary antibody overnight at 4°C. The following primary antibodies were utilized: Anti-Ad5 Capsid at 1:5,000 (Abcam, ab6982), E1A at 1:500 (BD Biosciences, 554155), DBP at 1:500 (hybridoma supernatant, mouse clone B6-8, original from A. Levine, kind gift of M. Weitzman), E4orf6 at 1:500 (hybridoma supernatant, mouse clone RSA3, original from D. Ornelles, kind gift of M. Weitzman), E1B55K at 1:500 (mouse clone 58K2A6, original from A. Levine, kind gift of M. Weitzman), E4orf3 at 1:500 (mouse, original from T. Dobner, kind gift of M. Weitzman), and GAPDH at 1:2,000 (LiCor, 926-42216). Membranes were washed three times with 1× TBST (Tris-Buffered Saline with Tween 20; 10 mM TrisHCl, 0.01% Tween 20, 150 mM NaCl in MilliQ Water), then anti-rabbit (LiCor, 926-68171), anti-mouse (LiCor, 827-08364), or anti-rat (Invitrogen, A48270) fluorescent secondary antibodies at 1:10,000 in TBST were applied to the membranes for a period of two hours. Membranes were washed three more times with 1× TBST and scanned on a LiCor Odyssey M.

### Fluorescent imaging

A549 cells were infected with WT, E3mNG, or E3gL5s at an MOI of 10. To compare fluorescent reporters, wells were simply imaged on an EVOS (Thermo Fisher) at 16, 24, or 48 hpi in the presence of Hoechst (Thermo Fisher R37605). Separately, A549s were infected with E3gL5s (MOI 10), treated with either DMSO, Hydroxyurea (1 mM), or Flavipiridol (300 nM) at 5 hours post-infection, and imaged at 24 hours post-infection.

### qPCR

Samples were lysed and purified using the GeneJET Genomic DNA Purification Kit (Thermo Fisher Scientific, K0721). qPCR was performed by combining 5 ng of DNA, primers, and 2× PowerUp SYBR Green Master Mix (Applied Biosystems, A25742) in a 12 μL reaction. Replication fold change was calculated with ddCT of viral DBP targets normalized to a Tubulin control. All primer sequences are found in Table S1.

### CellCyte live cell imaging

A549 cells were seeded in a single monolayer at 80%–90% confluence in black 96-well plates (Greiner, 655090) and infected with dual-reporter E3gL5s at an MOI of 10 in the presence of drug treatments included DMSO, 1 mM hydroxyurea (Cayman Chemical, Item No. 23725), or 300 nM flavopiridol (Selleck S1230), and were initiated 6 hours post-infection prior to imaging. Using a 4× objective and four images per well, green and red fluorescence were captured every hour for 48 hours and analyzed using CELLCYTE X software to threshold and determine the total number of fluorescent objects per well.

### Secreted luciferase assay

A549 cells were infected with either WT AdV or E3nLuc (MOI 10), and infection proceeded for the specified time. Supernatants or lysates were immediately read out for bioluminescence using the Nano-Glo Luciferase Assay System (Promega, N1110). Luciferase activity was measured via bioluminescence in relative light units (RLU). For reinfection assays, cleared lysates were diluted and used to reinfect cells for 6 hours, after which supernatants were collected and readout for enzyme activity as described above.

### Flow cytometry

Viral infections were performed at either MOI 1, 10, or 100 for a period of 48 hours. The panel of cell lines used was AD-293, HeLa, A549, Vero, and W162. At the appropriate time point, samples were rinsed with 1× PBS, trypsinized, and resuspended in 10% vol/vol DMEM, rinsed again, and finally resuspended in 2% FBS in PBS. Samples were processed on a FACSymphony A3 Cell Analyzer (BD Biosciences) and analyzed using FlowJo Software (BD Biosciences).

### Statistics

Statistics were performed using GraphPad Prism (v10.4.1). Statistical significance was calculated using an unpaired, two-tailed, standard t-test assuming Gaussian distribution and similar standard deviations. Linear regression was performed by calling the Simple Linear Regression formula using standard settings to approximate best-fit lines for the log-normalized data. Nonlinear regression was performed by calling the Specific Binding with Hill Slope formula with Least Squares Fit and standard settings. Where applicable, best-fit formulas and R^2^ values were provided.

## Data Availability

All raw data from this study are available from the corresponding author upon request. Plasmids and plasmid maps will be deposited with the Addgene repository.

## SUPPLEMENTAL MATERIAL

Table S1 jvi.01146-25-s0001.xlsx Oligonucleotide sequences used in this study.

ASM does not own the copyrights to Supplemental Material that may be linked to, or accessed through, an article. The authors have granted ASM a non-exclusive, world-wide license to publish the Supplemental Material files. Please contact the corresponding author directly for reuse.
